# Extended Duration Infusion of Hydroxocobalamin for Vasoplegic Rescue in Septic Shock

**DOI:** 10.7759/cureus.13388

**Published:** 2021-02-17

**Authors:** Harrison J Gerdes, Troy G Seelhammer, Scott Nei, Juan Diaz Soto, Christoph G Nabzdyk

**Affiliations:** 1 Anesthesiology and Perioperative Medicine, Mayo Clinic, Rochester, USA; 2 Pharmacy, Mayo Clinic, Rochester, USA

**Keywords:** hydroxocobalamin, hcb, septic shock, cyanokit, vasoplegia, nitric oxide, nos

## Abstract

Nitric oxide (NO) is a gaseous signaling molecule and a key endogenous mediator of vascular tone. Hydroxocobalamin (HCB) affects NO-mediated vasoplegia as (1) a direct inhibitor of nitric oxide synthase (NOS), thereby decreasing its production, and (2) by binding directly to NO and acting as a scavenger. HCB has been increasingly used in the treatment of refractory vasoplegia, particularly in cardiac surgery and liver transplant patients. Sepsis and septic shock are characterized by an increase in inducible NOS expression and activity with excessive NO production, resulting in endothelial dysfunction and profound systemic vasodilation. Therefore, a careful sustained reduction in NO burden represents a potential therapeutic target. Here, we present a case of refractory septic shock, which resolved after an extended duration infusion of high-dose HCB. We hope to foster further exploration regarding the safety, dosing, and efficacy of HCB when administered for vasopressor refractory septic shock.

## Introduction

Sepsis is defined as a life-threatening organ dysfunction caused by a dysregulated host response to infection and is characterized by a dramatic increase in inducible nitric oxide synthase (NOS) expression and activity with excessive production of nitric oxide (NO) [1,2]. As a result, sepsis leads to endothelial dysfunction and profound systemic vasodilation with associated hypotension which can have deleterious effects [3,4]. Therefore, a careful, sustained reduction in NO burden represents a potential therapeutic target. Hydroxocobalamin (HCB), which has traditionally been used in the treatment of cyanide toxicity, affects NO-mediated vasoplegia by (1) directly inhibiting NOS, decreasing NO production, and (2) by binding directly to NO and acting as a scavenger [5-8]. HCB has been successfully used in the treatment of refractory vasoplegia, particularly in cardiac surgery and liver transplant patients [9-11], but has not been extensively explored in septic shock. Shapeton et al. comprehensively summarized the pharmacology, toxicology, and clinical evidence surrounding the use of HCB in vasoplegic patients [5]. In this case report, we describe how a single extended duration infusion of high-dose HCB facilitates the resolution of catecholamine-refractory septic shock.

## Case presentation

A 73-year-old male with a 30-pack-year tobacco smoking history and poorly differentiated T4N0M0 right upper lobe lung adenocarcinoma status post-neoadjuvant chemotherapy underwent a right upper lobectomy. His comorbidities included type 2 diabetes mellitus, hyperlipidemia, and prostate cancer status post-radiation therapy and radical prostatectomy. He was discharged on postoperative day (POD) 12 but was re-admitted on POD 16 in the setting of a right-sided empyema and septic shock. After administration of empiric antibiotics, the patient was taken to the operating room for right thoracotomy with decortication. His intraoperative course was complicated by poor gas exchange with profound hypoxemia (arterial pO_2_/fraction inspired O_2_ or P/F ratio = 50; ref >300) and hypercarbia (partial pressure of carbon dioxide = 100 mmHg). In the context of this new-onset acute respiratory distress syndrome (ARDS) with poor compliance and associated elevated driving pressures (plateau pressure = 37 cmH_2_0, positive end-expiratory pressure = 12 cmH_2_0), the patient suffered an intraoperative, contralateral tension pneumothorax that required emergent needle decompression and subsequent left-sided chest tube placement. Intraoperatively, the patient required escalating doses of epinephrine, norepinephrine, and vasopressin to support hemodynamics. An intraoperative transthoracic echocardiogram (TTE) was performed, which revealed right ventricular (RV) dysfunction with an underfilled and hyperdynamic left ventricle (LV). These compounding factors resulted in a severe respiratory and metabolic acidosis with a pH of 7.1 at the time of arrival to the intensive care unit (ICU).

Upon admission to the ICU, initial resuscitation was aimed at correcting the respiratory and metabolic acidosis and improving RV performance. On arrival, the patient received tromethamine and, due to the severity of his ARDS, was paralyzed with cis-atracurium to optimize ventilator synchrony. Concomitantly, inhaled NO at 20 ppm was initiated to improve hypoxemia and decrease RV afterload. Within the first hour following these interventions, the patient’s acidosis had improved (pH = 7.32), as well as his hypercarbia and hypoxemia (PaCO_2_ = 50, PaO_2_ = 55). Four hours later, these were corrected (pH = 7.43, PaCO_2_ = 44, PaO_2_ = 72). At that time, serial bedside TTE showed normalization of RV function with persistence of a hyperdynamic LV. Central venous gas obtained three hours after admission to the ICU showed an SvO_2_ of 61.7%. Using concomitantly drawn arterial blood gas, this yielded a cardiac index (CI) of 3 L/min/m^2^ calculated by Fick. During the initial five hours of resuscitation, the patient received 2 L of crystalloid and 1 L of 5% albumin. His lactate peaked at 8 mmol/L on the third hour after ICU admission and normalized to 1.7 mmol/L eight hours later.

**Figure 1:**
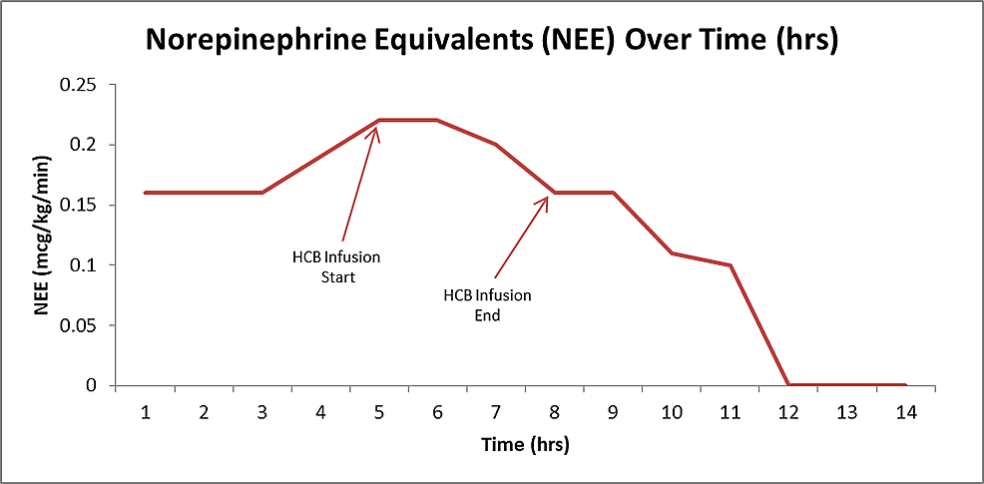
Norepinephrine equivalents over time (hours).

Despite normalization of his cardiac function (by bedside TTE and calculated CI), the patient continued to require high-dose infusions of norepinephrine (up to 0.09 mcg/kg/min), vasopressin (up to 0.04 units/min), and epinephrine (up to 0.1 mcg/kg/min), suggestive of vasodilatory shock. His vasopressor requirement did not improve after administration of stress dose steroids. Indeed, 23 hours after admission to the ICU, repeat central venous SatO_2_ was 73.9% for a calculated CI of 6 L/min/m^2^ by Fick. To address the refractory shock, he was given a one-time sustained infusion of HCB (5 g) administered over four hours. This resulted in a rapid, complete, and definitive weaning from all vasoactive agents over the ensuing 14 hours (Figure 1).

Two days later, he underwent re-exploration of the right thorax with right lung decortication, placement of additional right and left-sided chest tubes, and chest closure with negative pressure wound therapy device placement. Due to his overall weakened status after repeated interventions, he underwent elective tracheostomy and was transferred from the ICU to the respiratory care unit for ventilator weaning on POD 25 from the initial lobectomy. His chest tubes were removed and he was discharged from the hospital to a long-term acute-care facility on POD 42. Standard Health Insurance Portability and Accountability Act documentation and consent for use of the patient’s medical record for research purposes were obtained from the patient in accordance with the institutional policy.

## Discussion

Vasoplegia during sepsis can lead to poor patient outcomes. While patients with severe sepsis have mortality in excess of 30%, mortality for catecholamine-refractory septic shock requiring high-dose vasopressors (≥ 1 μg/kg/min of norepinephrine equivalents, calculated by summing norepinephrine-equivalent infusion rates of all vasopressors) has been reported to be approximately 80% [12,13]. The use of HCB in the setting of vasoplegia has been shown to improve hemodynamics and reduce vasopressor requirements in various case reports and case series, particularly after cardiopulmonary bypass and liver transplantation [14-16]. Importantly, no randomized trials have evaluated HCB for the management of vasoplegia. HCB has been proposed as a component of the therapeutic arsenal for the management of refractory vasodilatory shock where it is relegated for use as a rescue therapy along with methylene blue [17]. In the setting of sepsis, data are limited to a previous case report in which HCB was successfully used for the management of vasopressor refractory septic shock in two patients [18]. This case adds to that literature and suggests extending the dosing over four hours instead of a bolus [15,19].

Excessive NO availability in sepsis can cause hypotension and end-organ damage. Decreasing NO production and the secondary NO burden in septic shock patients appears to be a physiologically grounded therapeutic target [2]. NO is a gaseous signaling molecule and a key endogenous mediator of vascular tone. NO is produced by several NOS isoforms, whose activities are markedly different during states of health and systemic disease [4]. Unregulated, excessive NO production by inducible NOS in vascular smooth muscle cells has been postulated as a mechanism for the derangement in vascular tone present in septic shock [4]. Unfortunately, direct pharmacologic NOS inhibition through a fixed drug dose regimen failed to yield benefits in a population of septic shock patients and, in fact, resulted in increased mortality in one placebo-controlled, multicenter randomized trial [2]. This might imply that careful titration of NO-depleting drugs might be more appropriate, rather than a one-size fits all approach.

Lin et al. described two patients who received high-dose HCB as a rescue agent during distributive septic shock [18]. Similar to what we observed, both patients experienced a significant improvement in arterial pressure, which allowed the rapid down-titration of vasopressor support. Of note, we used the same dose as previously reported (5 g), but in our case, this was administered as an extended infusion over four hours. An extended duration infusion was utilized because a previous report described the duration of hemodynamic effect from a bolus lasting about 210 minutes [9], while the literature demonstrates positive hemodynamic effects extending to 600 minutes with the extended duration infusion [15,19]. Therefore, as previously mentioned, to sustain the desired effects beyond 210 minutes (observed for 19 hours in this case), the dose was extended over four hours. The drug has a beyond use time of six hours after mixture in the manufacturer bottle, so four hours was chosen to complete the infusion within the six-hour timeframe. When examined together, these cases invite further exploration regarding the efficacy of HCB in the setting of vasopressor refractory septic shock, while reinforcing the need for additional dose-finding and safety trials.

This report supports previous literature and advocates for an extended duration infusion of high-dose HCB, which may offer an advantage by attenuating the prolonged NO dysfunction encountered in sepsis. This strategy was chosen because in a previous study of post-cardiac surgery vasoplegia, the beneficial hemodynamic effects only lasted 210 minutes after a bolus [9], but lasted 600 minutes with an extended duration infusion [15,19]. In sepsis, NO elicits both beneficial and deleterious effects on various organ systems, which are not limited to a short timeframe such as during cardiopulmonary bypass cases [20]. Therefore, it may be prudent to not completely and non-selectively abrogate NO production in sepsis for a short time only (bolus). Instead, it might be beneficial to mitigate excess NO abundance to help restore vascular tone in profoundly hypotensive patients via a slow and controlled infusion of a NO scavenger and NOS inhibitor such as HCB [5]. It is conceivable that a prolonged infusion of HCB may better match the patient’s clinical state of enhanced NO production during sepsis. The manufacturer states that the beyond use time of the drug after mixing is six hours, so a clinical choice based on previous literature and theory was made to extend the drug delivery over four hours. The extended duration HCB dosing strategy produced a sustained response in this case and warrants further investigation.

## Conclusions

Sepsis and septic shock are characterized by a dramatic increase in inducible NOS expression and activity with excessive NO production. As a result, sepsis leads to endothelial dysfunction and profound systemic vasodilation. HCB improves NO-mediated vasoplegia by inhibition of NOS and by directly binding to NO, thereby acting as a scavenger. HCB has been successfully used in the treatment of refractory vasoplegia from different etiologies but is yet to be explored in the setting of sepsis. Here, we presented a case of refractory septic shock which resolved with a single extended duration infusion of HCB. Considering the potential therapeutic benefits of HCB in the setting of sepsis pathophysiology, the absence of a dose/administration finding study, and an as-yet unknown safety profile, we propose a call to arms for further investigations into the use of HCB for vasopressor refractory septic shock.
